# Weather and Seasonality Associations With Vestibular Neuritis

**DOI:** 10.7759/cureus.97025

**Published:** 2025-11-17

**Authors:** Athanasios Vlachodimitropoulos, Gerasimos Danielides, Theodoros Stathas, Georgios Batsaouras, Vasileios Papanikos, Christine Dafni, Spyridon Lygeros

**Affiliations:** 1 Otolaryngology - Head and Neck Surgery, University General Hospital of Patras, Patras, GRC; 2 Pharmacy, University General Hospital of Ioannina, Ioannina, GRC

**Keywords:** climate, environmental factors, heat stress, seasonality, temperature, vestibular disorders, vestibular neuritis

## Abstract

Background: Vestibular neuritis (VN) is an acute vestibular syndrome often attributed to viral or post-viral inflammation, though evidence for seasonal or environmental triggers remains inconsistent. While early studies suggested spring or summer peaks, more recent data have questioned whether VN incidence follows a clear seasonal pattern. Beyond broad seasonality, specific meteorological factors may influence VN occurrence.

Objective: This study aims to investigate seasonal variation and associations between VN incidence and local weather anomalies in a Mediterranean climate.

Methods: We retrospectively reviewed VN cases presenting to a tertiary hospital in Patras, Greece, between January 2020 and September 2025. Diagnoses were based on Bárány Society criteria. Daily meteorological data (temperature, wind speed, atmospheric pressure) were obtained from Meteostat and aggregated by month. We assessed seasonality with a chi-square goodness-of-fit test and calculated Pearson correlations between monthly VN cases and weather anomalies (deviations from long-term monthly means). Bonferroni correction was applied for multiple testing.

Results: A total of 102 VN patients were identified (mean age, 54.7 ± 15.4 years; 58 (56.9%) female cases). No significant seasonality was observed in monthly case distribution (χ² = 14.58, p = 0.2). However, maximum temperature anomalies were positively correlated with VN cases (r = 0.39, p = 0.0009; Bonferroni-adjusted p = 0.0047). Wind speed and average temperature anomalies demonstrated a negative and positive correlation with VN cases, respectively (p < 0.05), which did not reach significance after Bonferroni correction (p > 0.01). Other meteorological variables showed no significant associations.

Conclusion: The incidence of VN in our cohort did not follow a strict seasonal pattern but was significantly associated with higher-than-average monthly maximum temperatures. This supports a multifactorial pathogenesis in which viral reactivation, vascular compromise, and immune dysregulation may interact with environmental stressors. These findings highlight the importance of considering climatic influences in VN and warrant further multicenter, high-resolution studies integrating virological and environmental data.

## Introduction

Vestibular neuritis (VN) is an acute vestibular syndrome characterized by the sudden onset of severe vertigo, nausea, and imbalance without hearing loss [1]. It is thought to result from dysfunction of the vestibular nerve, often presumed to be due to a viral infection or post-viral inflammatory process [1]. Indeed, many patients report an upper respiratory infection or "viral prodrome" preceding the vertigo onset [2]. The annual incidence of VN is relatively low (around 3.5 per 100,000 population) [3], but VN accounts for a significant subset of acute dizziness cases in ENT and neurology practice. If a viral etiology is correct, one might expect seasonal patterns in VN occurrence, as many viruses show seasonal cycles (for example, influenza in winter [4]). Early descriptions suggested VN cases tended to occur in spring and early summer [5], paralleling some viral infection peaks. Histopathologic studies have also noted vestibular nerve degeneration in VN similar to that seen in viral inner ear diseases such as mumps [1], lending plausibility to the viral hypothesis.

However, the evidence for seasonality in VN has been mixed. Recent studies have failed to demonstrate any significant seasonal variation in VN incidence [5-7]. On the other hand, some clinicians maintain that VN can cluster in certain months [1], so the question remains controversial. Moreover, beyond broad seasons, specific weather conditions (temperature, atmospheric pressure, and others) could theoretically influence VN incidence, for instance, via effects on viral transmission or on the inner ear’s homeostasis (e.g., barometric pressure changes on labyrinthine fluid). To our knowledge, the relationship between VN and specific meteorological variables has not been well characterized.

The aim of this study was to investigate the seasonality of VN and its potential associations with weather factors. A retrospective analysis of VN cases over a recent five-year period was conducted. First, VN cases were examined to determine whether they were distributed evenly throughout the year or showed seasonal peaks. Then, correlations between VN case frequencies and local weather parameters such as temperature, wind, and atmospheric pressure were analyzed using an anomaly-based approach to adjust for normal seasonal fluctuations. By integrating clinical data with meteorological data, we sought to clarify whether VN occurrence is truly random throughout the year or if subtle environmental influences can be detected. This could provide insight into VN’s underlying causes, for example, supporting a viral hypothesis if a winter respiratory virus pattern emerged or suggesting other mechanisms if non-viral weather correlations were found. Ultimately, understanding any temporal or environmental triggers for VN could improve our understanding of its pathogenesis and help anticipate case patterns or triggers in susceptible patients.

## Materials and methods

Study design and setting

A retrospective observational study of VN cases presenting to a tertiary hospital in Patras, Greece, was performed over a period of approximately 5.75 years. The study period was from January 1, 2020, through September 30, 2025. Patras is in a Mediterranean climate zone with mild, wet winters and hot, dry summers, which provides a broad range of weather conditions for analysis. All patients included were from the hospital’s Otolaryngology services, serving a regional population. The hospital’s ethics committee approved the data collection and analysis (approval no. 23111).

Inclusion criteria

All patients who were diagnosed with VN during the study period were included. The diagnosis was made by ENT specialists based on the diagnostic criteria of the Bárány Society for VN: acute or subacute onset of sustained spinning or non-spinning vertigo of moderate to severe intensity with symptoms lasting for at least 24 hours, spontaneous peripheral vestibular nystagmus with a trajectory appropriate to the semicircular canal afferents involved (generally horizontal-torsional, direction-fixed, and enhanced by removal of visual fixation), unambiguous evidence of reduced vestibulo-ocular reflex (VOR) function on the side opposite the direction of the fast phase of the spontaneous nystagmus, no evidence of acute central neurological, otological, or audiological symptoms, and no acute central neurological signs [8]. Patients with findings suggestive of benign paroxysmal positional vertigo (BPPV), Ménière’s disease, or labyrinthitis (vestibular labyrinth involvement with hearing loss) were excluded. Evidence of reduced VOR function was established with a positive head impulse test (HIT).

Data collection

For each VN case, the date of presentation and patient demographics (age and sex) were recorded. Meteorological data were sourced from the Meteostat API using the hospital’s geographic coordinates (latitude 38.2411°, longitude 21.7350°). Daily meteorological measurements, including average temperature (°C), minimum and maximum temperature (°C), average wind speed (m/s), and mean sea-level atmospheric pressure (hPa), were collected. These daily weather variables were merged with the daily patient counts. For analysis purposes, data were aggregated by month to assess seasonality, as some weather variables (e.g., temperature) vary more discernibly by month.

Statistical analysis

First, the data were described using summary statistics. Patient characteristics are reported as counts and percentages for categorical variables and as mean ± standard deviation (SD) for age. The weather variables studied are summarized over the 2020-2025 period.

Next, to assess the seasonality of VN cases, the total number of cases in each month of the year from January 2020 through December 2024 was tabulated. The distribution of cases across the 12 months was evaluated to determine whether it was significantly different from a uniform distribution using a chi-square goodness-of-fit test. A p-value < 0.05 was considered statistically significant for this seasonality test.

For the weather correlation analysis, an anomaly method was employed to adjust for normal seasonal variation. Specifically, for each calendar month, the average of each weather variable was calculated. Then, monthly "weather anomalies" were defined as the deviation of each month’s value from the average for that month. Thus, each monthly interval from January 2020 to September 2025 received an anomaly value for all weather variables studied.

After adjusting for seasonal variation, the Pearson correlation coefficient (r) between the monthly number of VN cases and each weather variable for the entire period (January 2020 through September 2025) was computed. Given that multiple weather factors were tested, a Bonferroni correction was applied to adjust for multiple comparisons and avoid false-positive findings. P values < 0.05 were considered statistically significant for correlation testing, whereas p values < 0.01 were considered significant after Bonferroni correction. All statistical tests were conducted using Python 3.11, with libraries including pandas, numpy, scipy, matplotlib, and seaborn. All p-values are two-tailed.

## Results

Patient characteristics

Over the study period, a total of 102 patients were diagnosed with VN (from January 2020 to September 2025). Of these, 84 cases occurred in the five-year span from 2020 to 2024, which was used for the seasonality analysis (the remaining 18 occurred in the first nine months of 2025). The patients had a mean age of approximately 54.7 years (SD = 15.4, range 16 to 82 years). Fifty-eight (56.9%) patients were female, and 44 (43.1%) were male (Table 1).

**Table 1:** Characteristics of Vestibular Neuritis Cases (2020–2025)

Variable	Value
Total cases	102
Age (mean ± SD, range)	54.7 ± 15.4 (16-82)
Female sex	58 (56.9%)

Seasonality of VN cases (2020-2024)

A total of 84 cases occurred in this five-year period, averaging approximately 1.4 cases per month. August had the highest number of cases (12 cases over five years, representing 14.3% of the total), while March had the fewest (2 cases, 2.4% of the total). In general, late spring and summer months (May and August) had slightly elevated case counts, whereas late winter and mid-summer (March and July) had lower counts (Table 2). However, these differences did not reach statistical significance (χ² = 14.58, p = 0.2).

**Table 2:** Monthly Distribution of Vestibular Neuritis Cases (2020–2024)

Month	Number of Cases	Percentage of Total (%)
January	9	10.7
February	6	7.1
March	2	2.4
April	6	7.1
May	11	13.1
June	8	9.5
July	3	3.6
August	12	14.3
September	6	7.1
October	4	4.8
November	9	10.7
December	8	9.5

Correlation of VN cases with weather variables

A total of 69 monthly intervals (January 2020 to September 2025) were analyzed (Table 3).

**Table 3:** Summary of Meteorological Variables (2020–2025)

Variable	Mean ± SD
Average temperature (°C) (tavg)	18.66 ± 6.36
Minimum temperature (°C) (tmin)	14.85 ± 5.85
Maximum temperature (°C) (tmax)	23.00 ± 6.94
Atmospheric pressure (hPa) (pres)	1015.73 ± 3.48
Wind speed (m/s) (wspd)	7.89 ± 1.34

Pearson correlation tests were performed between monthly VN case counts and anomalies of five weather variables (tavg, tmin, tmax, wspd, pres), calculated as deviations from the long-term monthly mean (Table 4).

**Table 4:** Monthly Mean Meteorological Parameters (2020–2025)

Month	Average Temperature (°C) (tavg)	Minimum Temperature (°C) (tmin)	Maximum Temperature (°C) (tmax)	Atmospheric Pressure (hPa) (pres)	Wind Speed (m/s) (wspd)
January	10.83	7.90	14.68	1019.28	8.32
February	11.23	7.97	15.36	1020.48	8.09
March	12.46	8.95	16.64	1016.97	8.57
April	15.38	11.33	19.98	1014.66	9.22
May	19.97	15.87	24.43	1014.60	8.23
June	25.04	20.38	29.55	1013.33	7.76
July	28.31	23.39	33.27	1011.21	7.88
August	27.56	23.04	32.45	1011.33	7.33
September	23.94	19.77	28.72	1014.59	7.38
October	19.48	15.69	23.97	1018.21	6.86
November	15.83	13.01	19.41	1017.81	7.34
December	12.53	9.95	15.88	1017.37	7.32

**Figure 1:**
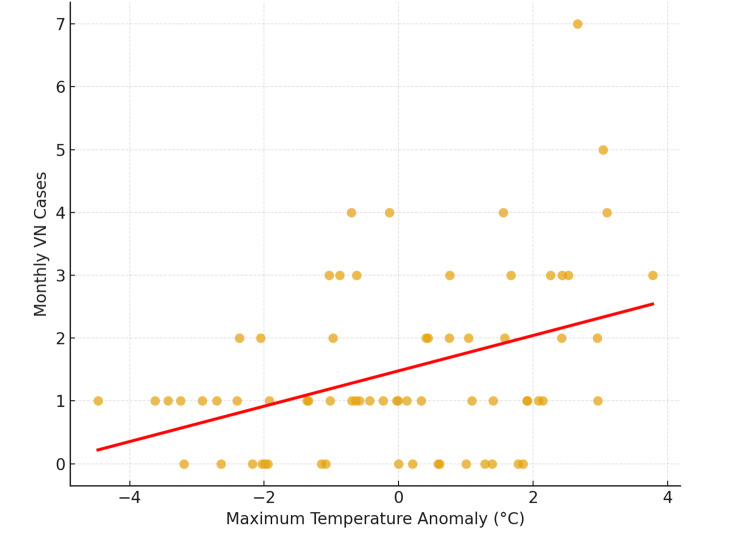
Monthly Vestibular Neuritis Cases vs Monthly Maximum Temperature Anomalies (2020–2025) VN: vestibular neuritis.

Maximum temperature anomaly (tmax-a) showed a statistically significant positive correlation with VN case counts (r = 0.39, p = 0.0009). This association remained significant after Bonferroni correction for multiple comparisons (adjusted p = 0.0047) (Table 5, Figure 1). Wind speed anomaly (wspd-a) and average temperature anomaly (tavg-a) demonstrated negative and positive correlations with VN cases, respectively (p < 0.05), but these did not reach significance after Bonferroni correction (p > 0.01). Minimum temperature anomaly (tmin-a) and pressure anomaly (pres-a) showed no significant correlation (Table 5).

**Table 5:** Correlation Between Vestibular Neuritis Cases and Meteorological Variables (2020–2025) Pearson's r: Pearson's correlation coefficient; p-value: p-values < 0.05 are considered statistically significant; Bonferroni-adjusted p: p-values < 0.01 are considered statistically significant; *: non-significant p-values.

Variable	Pearson’s r	p-value	Bonferroni-Adjusted p
Average temperature anomaly (°C) (tavg-a)	0.302	0.0116	0.0581*
Minimum temperature anomaly (°C) (tmin-a)	–0.041	0.7369*	10.000*
Maximum temperature anomaly (°C) (tmax-a)	0.390	0.0009	0.0047
Atmospheric pressure anomaly (hPa) (pres-a)	–0.055	0.6522*	10.000*
Wind speed anomaly (m/s) (wspd-a)	–0.271	0.0243	0.1215*

## Discussion

The absence of a significant seasonal pattern in VN in our data aligns with the growing consensus in recent research. Earlier hypotheses postulated a spring or summer peak for VN (presumably due to viral causes) [9], but most empirical studies have failed to confirm a strong seasonal concentration. Our results corroborate those of Koors et al. (2013), who found only "minimal evidence" of seasonality in VN cases over 36 months [6]. Similarly, Jeong et al. (2022) reported no significant monthly or seasonal differences among 248 VN patients in Korea, despite a slight numerical peak in July [9]. A large German analysis also showed uniform VN incidence across seasons [9]. In Brazil’s tropical climate, Bilecki et al. (2005) also concluded there was no seasonal correlation for vestibular disorders, including VN [10]. Taken together, these studies, along with our own, strongly suggest that VN is not tied to a particular season in the way that many viral illnesses are. This finding has important implications. It suggests that if viruses are involved in VN, it may not be a single seasonal virus or a narrow window of viral outbreaks. Instead, VN might be caused by a variety of pathogens that strike at different times, or by ubiquitous viruses (such as reactivations of Herpes Simplex Virus (HSV) type 1 [11]) that are not season-dependent. It also raises the possibility that non-viral factors play a role in precipitating VN.

Recent research in the past decade has indeed highlighted non-viral triggers for VN, including vascular, immune-mediated, and stress-related factors. On the vascular side, mounting epidemiological evidence links VN with cardiovascular risk factors. Oron et al. (2017) found that patients hospitalized with VN had a higher prevalence of hypertension, diabetes, and dyslipidemia compared to controls [12]. This aligns with Adamec et al. (2015)’s prospective study, which reported that a significant proportion of VN patients over 50 had hypertension (30%), diabetes (9%), or hyperlipidemia [5]. Such comorbidities could predispose to microvascular compromise of the inner ear. Indeed, an inflammatory thrombogenic process in the anterior vestibular artery has been proposed: VN patients show elevated peripheral monocyte activation (CD40-positive monocytes/macrophages) and cytokines that can promote microthromboses, leading to reduced perfusion of the vestibular organ [13]. This small-vessel ischemic injury to the vestibular nerve or labyrinth could produce an acute vestibular syndrome clinically indistinguishable from viral neuronitis.

Autoimmune or immune-mediated pathways represent another non-viral etiologic avenue. An influential review by Greco et al. (2014) asks whether VN could be an immune-related neuropathy analogous to a localized Guillain-Barré or multiple sclerosis (MS) affecting the vestibular nerve [2]. Support for this idea comes from immunological findings: patients with VN can exhibit an imbalance between T-helper and T-suppressor cells similar to that seen in MS [14]. There are case reports of demyelinating lesions (MS plaques) selectively involving the vestibular nerve and mimicking VN [2], implying that autoimmunity can selectively target this system. It is conceivable that in some instances a viral infection serves as a trigger for an autoimmune reaction against vestibular nerve epitopes, or that idiopathic autoimmunity arises, causing VN without any active infection. The efficacy of corticosteroids in VN treatment, which may improve peripheral vestibular recovery, whereas antivirals alone do not [15], is consistent with an inflammatory or immune-mediated injury as the final common pathway.

Stress-related mechanisms also deserve mention. "Stress" in this context can be physiological (heat or exertion) or psychological. Recent studies have revealed a robust relationship between stress (both physiological and psychological) and symptomatic HSV recurrence [16], which has been linked to the onset of VN [17]. Additionally, stress elevates cortisol and catecholamine levels [18], which might transiently reduce inner ear blood flow or modulate immune responses, compounding the risk of either a viral or ischemic event in the vestibular nerve. While direct studies on stress as a trigger for VN are lacking, the concept aligns with the multifactorial etiology that recent literature endorses.

The positive correlation of VN incidence with high temperature anomalies, coupled with a nonsignificant negative correlation with wind anomalies, can thus be interpreted in light of the above mechanisms. First, unusually hot weather constitutes a physiologic stressor that might precipitate reactivation of latent viruses implicated in VN (HSV type 1) or otherwise impair host immune responses [16,17]. Second, heat exposure can promote dehydration and hemoconcentration [19], potentially compromising the inner ear’s microcirculation. Higher temperatures have been linked to increased blood viscosity and cardiovascular strain, which in susceptible individuals could lead to labyrinthine ischemia (similar to how temperature or pressure shifts can trigger strokes or myocardial infarctions [19]), a proposed non-viral mechanism for acute vestibular dysfunction [14].

In our analysis, higher-than-average wind speeds showed a slight negative correlation with VN case frequency, but this trend was not statistically significant. There is little direct literature on wind as an isolated trigger for vestibular disorders, which is consistent with the absence of a significant effect in our data. Indirectly, windiness often accompanies specific weather systems that include pressure and temperature changes, so any influence of wind could be confounded by those factors. One speculative hypothesis is that strong winds disperse environmental pollutants or viral particles and thus might reduce exposure to certain triggers. Another possibility is behavioral, for instance, very windy or stormy conditions might reduce outdoor activities and close-contact gatherings, slightly decreasing transmission of respiratory viruses that can antecede VN. However, these ideas remain conjectural. Notably, the UK Ménière’s disease study monitored wind speed along with other variables, and it found that wind per se did not emerge as a significant predictor of symptom severity when pressure and humidity were accounted for [20]. Therefore, the mild negative association between wind anomalies and VN in our findings is likely incidental or mediated by other meteorological conditions.

We should acknowledge several limitations in our study. First, the sample size (84 cases for seasonality, 102 total) is moderate, sufficient to detect large effects but possibly underpowered for subtle patterns. A few more cases could tip a month’s count and change the outcome. Second, as an observational study, we can only report associations, not prove causation. Unmeasured confounders such as air pollution levels, viral infection rates, or individual patient comorbidities could underlie or modulate the observed weather relationships. Third, our analysis was ecological (monthly aggregate level), which is appropriate for detecting broad correlations but cannot prove causality at the individual level. We assumed that if a weather factor is a trigger, it would show up in aggregate timing of cases, but individual susceptibility varies. Fourth, our study is from a single geographic region, and results might differ in other climates. Finally, meteorological data were taken from area-wide stations, and individual patients’ true exposures might differ (for example, indoor climate control or travel could modify personal exposure to ambient conditions).

## Conclusions

In this study, we found no evidence of strict seasonality in vestibular neuritis incidence, but we identified a significant positive correlation between monthly maximum temperature anomalies and case frequency. This finding suggests that environmental stressors, particularly heat, may act as triggers for VN, potentially through viral reactivation, vascular compromise, or immune modulation. The absence of a clear seasonal pattern, combined with growing evidence of vascular and autoimmune contributors, underscores the multifactorial nature of VN. VN appears to arise from the interplay of infectious, vascular, and immune mechanisms influenced by environmental exposures. Clinically, awareness of heat-related susceptibility may help guide patient counseling during periods of extreme weather, particularly in older patients or those with vascular comorbidities. Future multicenter studies with larger sample sizes and daily-level weather linkage are warranted to clarify the role of specific meteorological triggers. Integrating meteorological data with clinical and virological markers may ultimately improve risk stratification and help predict case patterns in vestibular disorders.
